# Environmental Impact of Care for End-stage Kidney Disease on the Earth and Humans

**DOI:** 10.31662/jmaj.2021-0105

**Published:** 2021-12-03

**Authors:** Kei Nagai, Norihiro Itsubo

**Affiliations:** 1 Department of Nephrology, Hitachi General Hospital, Hitachi, Japan; 2 Department of Nephrology, Faculty of Medicine, University of Tsukuba, Tsukuba, Japan; 3 Faculty of Environmental and Information Studies, Tokyo City University, Yokohama, Japan

**Keywords:** hemodialysis, carbon footprint, water footprint, life cycle assessment

Hemodialysis for end-stage kidney disease is becoming increasingly necessary around the world, but it is an incredibly resource-intensive therapy. Therefore, the increased clinical use of hemodialysis is expected to have adverse effects on natural resources such as fossil fuels, forests, and animal life. Moreover, climate change associated with increased CO_2_ emissions is expected to threaten human health and the global economy. The carbon footprint (CFP) is a common index used to estimate the environmental burden of human activity. While investigations regarding the CFP of healthcare services have been promoted ^(1)^. few studies have quantitatively and comprehensively estimated the actual impact of hemodialysis on society.

Care for patients with end-stage kidney disease who require chronic dialysis therapy comes at a substantial cost in terms of waste and water and energy consumption. Water consumption is a major concern; therefore, some countries conduct water reuse practices when providing hemodialysis to help avoid water shortages ^(2)^. Water footprint (WFP) is an index that quantitatively measures the amount of water consumed in the process of production, consumption, and disposal of goods and products. A cup of coffee is about 200 ml, but 140 L of water is used to produce and consume the coffee beans needed to brew. Similarly, water consumption should cover every activity related to care for kidney disease, such as the development of medicines and the generation of electricity, as well as direct consumption via dialysate production (Figure 1A). However, to the best of our knowledge, no previous studies have assessed the WFP associated with hemodialysis, other than considering how to manage and reduce actual water consumption in a dialysis session ^(3)^. To promote public health, it would be informative to estimate the overall impact and environmental burden of hemodialysis on human health and the planet. Therefore, this study aimed to quantify the impact of hemodialysis based on a life-cycle assessment of the Earth and humans (Figure 1B).

**Figure 1.**
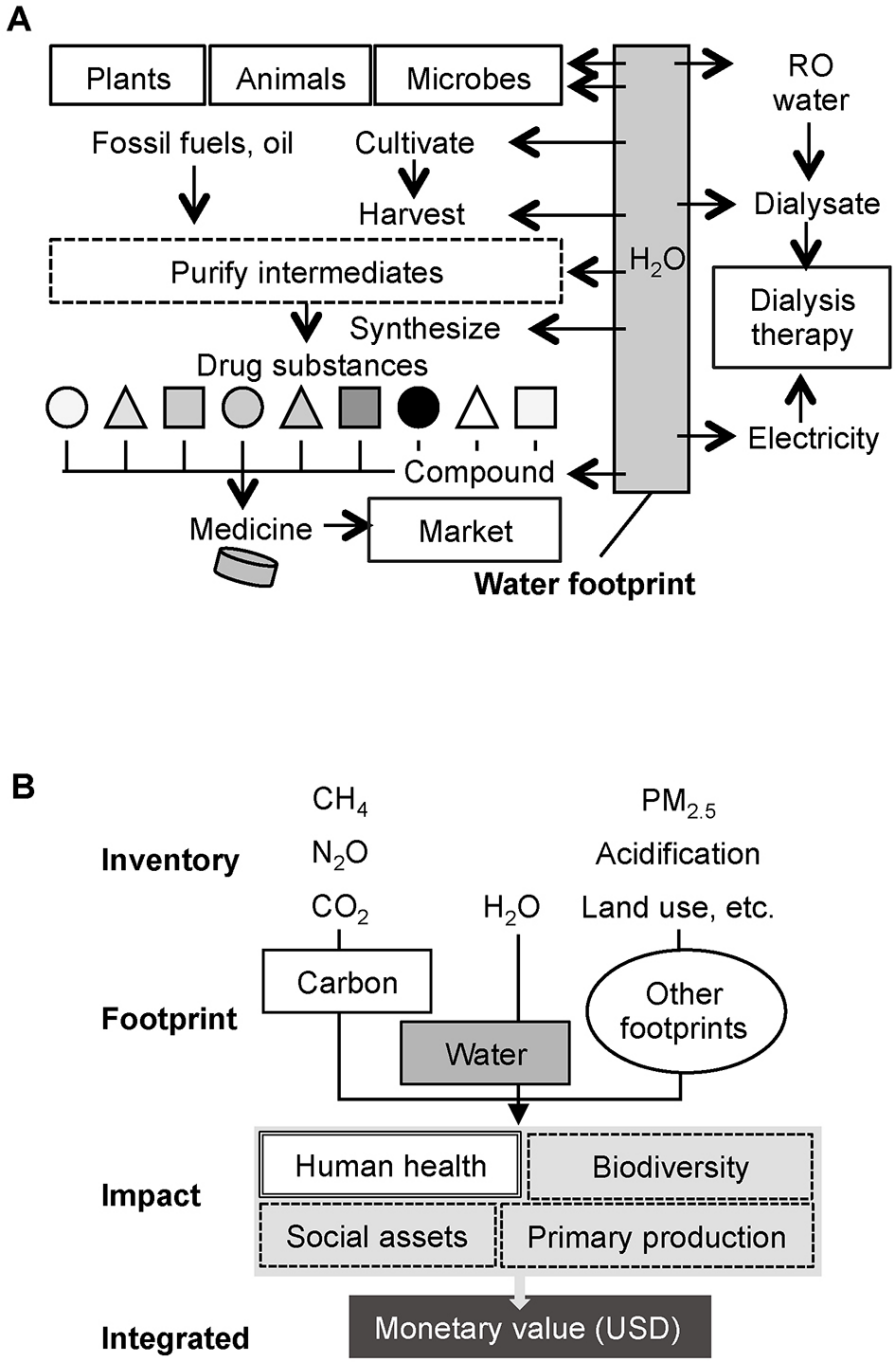
Framework of the water footprint study and life cycle impact assessment for dialysis therapy. A Dialysis therapy requires water for the pharmaceutical production and energy generation processes and for the production of reverse osmosis (RO) water and dialysate in a facility. Drugs are compounds of drug substances originating from natural resources such as plants, animals, and microbes. These steps require a certain amount of water both directly and indirectly. The water footprint exceeds the actual water consumption for dialysate in a dialysis session and hot water disinfection between sessions. B Carbon and water footprints were calculated by summing every inventory item multiplied by the amount used. Several impacts, including that on human health, can be drawn with proper coefficients in the Life Cycle Impact Assessment Method based on endpoint modeling. Finally, monetary value can be estimated by integrating the intensity of the impacts.

We utilized CFP inventory based on Embodied Energy and Emission Intensity Data for Japan Using Input-Output Tables provided by the Center for Global Environmental Research, National Institute for Environmental Studies ^(4)^. The Inventory Database for Environmental Analysis (IDEA) version 2 can present the overall WFP for numerous kinds of industrial products ^(5)^. In this study, we selected four items, namely, water use and waste, transportation, medicine, and dialysis machine and electricity. The price-based basic units for CFP and WFP were obtained from relevant inventories, and the CO_2_ equivalent (kg eCO_2_) and H_2_O m^3^ were calculated by multiplying the estimated price. In this study, the amount of medicine was 60.5 thousand yen and that of medical equipment was 45.0 thousand yen per person-year. Electricity use is 6.0 kWh per session. Water use and waste were 150 L/session, 32 km of transportation by a car per dialysis session, and 21.8 thousands yen for 1 year’s use of a dialysis machine for one patient. Most traditional integration methods for life cycle assessment were established based on “midpoint” modeling. However, these methods need to be improved because they compare impact categories without showing information on the degree of actual environmental “impact” in the end. Subsequently, the methodology of using the results of damage assessment called Life Cycle “Impact” Assessment (LCIA) rapidly attracted attention. In this study, we further applied the Life Cycle Impact Assessment Method based on endpoint modeling (LIME) ^(6)^, and it enables to estimate water consumption ^(7)^, human health damage factors ^(8)^, and monetary weighting factors ^(9)^. The factors of health damage for CO_2_ emissions and water are 4.19 ×10^-7^ disability-adjusted life years (DALYs) per kg eCO_2_ and 9.65 × 10^-6^ DALYs per H_2_O m^3^, respectively. The integration factors for CO_2_ emissions and water are 1.71 × 10^-2^ USD per kg eCO_2_ and 2.22 × 10^-1^ per H_2_O m^3^, respectively. All calculations were performed using commercially available software (SimaPro v8.1.1.22, TCO2 Co., Ltd., Tokyo, Japan).

This estimation adopts a model in which chronic hemodialysis treatment is given three times per week for one patient (4 h per treatment session) and in line with the current status of dialysis therapy in Japan ^(10)^. Medicine values include pharmaceuticals and any treatments shown in 1-year medical receipts for patients undergoing chronic hemodialysis ^(11)^. For this investigation, clinical engineers in dialysis facilities in Ibaraki, Japan, were interviewed regarding eco-friendly dialysis. Ethics approval was not sought since, in our judgment, there was no need to seek formal ethics clearance because the project did not collect personal data from patients or healthcare providers or expose either group to any risk, as in the previous study ^(12)^.

Based on our price-based estimations of water and electricity consumption, medical costs, and waste, the CFP of hemodialysis treatment is substantial (Figure 2A), consistent with a previous study ^(12)^. Our results suggest that the WFP of the procurement of medicine could exceed that of the water supply (Figure 2B). LIME can estimate the health impact using DALYs and the costs of environmental impacts, with damage as the endpoint. We calculated DALYs based on the sum of the coefficient adjusted-CFP and -WFP, estimated as 2.4 × 10^-3^ per person-year (Figure 2C). The financial burden, calculated in the same way, was 85.6 USD per person-year (Figure 2D).

**Figure 2.**
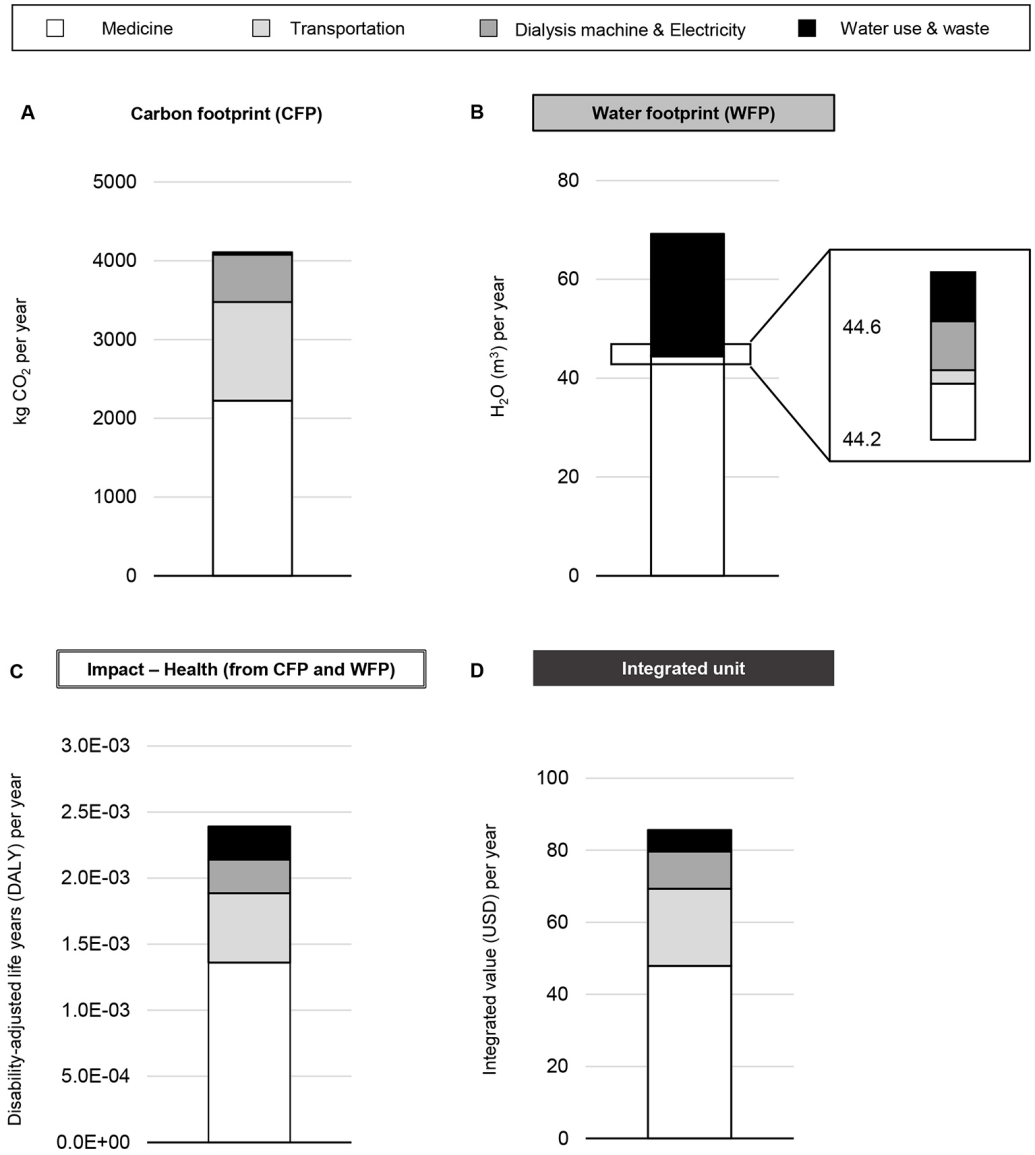
Results of the life cycle impact assessment for dialysis therapy. A, B The carbon and water footprints for hemodialysis are calculated to estimate the integrated impact of hemodialysis on the Earth and humans based on the Inventory Database for Environmental Analysis (IDEA) and Embodied Energy and Emission Intensity Data for Japan Using Input-Output Tables (3EID). C, D This estimation adopts a model in which chronic hemodialysis treatment is given three times per week for one patient. Human health impact and integrated monetary value are calculated using the carbon and water footprints and are likely underestimated due to study limitations.

Environmental problems are sometimes difficult for medical professionals to understand in the clinical setting. In daily practice, healthcare providers rarely consider the direct effect of greenhouse gas emissions on their patients. In turn, from a public health perspective, clinical physicians and allied healthcare providers may accept the identified risks regarding the health impact and financial burden caused by changes in the environment. Hemodialysis requires substantial resources of water and electricity in association with the production process for medicines and plastic. Life cycle assessment can help integrate all environmental impacts into a single index, such as one for human health and financial value in order that healthcare providers make out well ^(13)^.

It was surprising that the WFP for medicine might be the highest, followed by water use and waste related to the production of dialysate. This result implies that medicine is a major contributor to both WFP and CFP, which is compatible with a previous study reporting that pharmaceutical emissions were 35.7% and dominated the CFP of hemodialysis services ^(12)^. Therefore, minimization of medications and non-pharmaceutical interventions that have lower environmental costs are possible ways to reduce the WFP associated with hemodialysis.

Our finding is that hemodialysis for 1 patient-year costs 2.4 × 10^-3^ DALYs, which indicates that annual dialysis procedures for 418 patients could represent 1-year disability-adjusted losses. Similarly, hemodialysis costs 85.6 USD for 1 patient-year, which translates to an annual economic burden of 28 million USD in medical costs for 330,000 patients (i.e., the approximate number of Japanese patients receiving dialysis) besides medical expenses. To the best of our knowledge, this is the first attempt of evaluating WFP and LCIA regarding any other care for end-stage kidney disease, namely, peritoneal dialysis, renal transplantation, and conservative kidney management. As peritoneal dialysis and renal transplantation are expected to have an advantage on environment over hemodialysis mainly based on the evidences of CFP studies ^(14)^, WFP and LCIA should be studied for comparing superiority among them in the future.

Our model has many barriers to more precise integration. First, our analysis is dependent on the IDEA, which was nonspecifically developed by the industrial science and environmental management communities. Second, the CFP and WFP components were evaluated based on cost, not on actual use. Third, the information used in this study was obtained by inquiries sent to dialysis facilities. Therefore, a large field survey is needed to ensure the reliability of our findings.

Nevertheless, our proposed method could be a tool to help explain the adverse health effects and economic burdens associated with hemodialysis therapy itself. The remaining challenges include establishing relevant inventory databases for dialysis therapy and conducting multicenter patient-based cohort studies focused on dialysis materials and consumption. The ultimate goal of this work is to inform clinical physicians and allied healthcare providers of the importance of initiating more eco-friendly kidney care practices to improve global DALYs. Such a cost-based index could provide companies and politicians with more information regarding the potential cost reductions that could be achieved through eco-dialysis. This could lead to both policy and social changes toward resource-saving technology.

## Article Information

### Conflicts of Interest

None

### Sources of Funding

This study was supported by [the Japan Society for the Promotion of Science (JSPS)] [grant number #18KK0431] and [the Japanese Association of Dialysis Physicians] [grant number #2019-1].

### Acknowledgement

We would like to thank Hiroaki Suzuki and Atsushi Ueda for contributing to the data collection and the clinical engineers in the dialysis facilities in Ibaraki, Japan, for kindly agreeing to participate in interviews regarding eco-friendly dialysis.

### Author Contributions

Conceptualization, investigation, and writing - original draft preparation: Kei Nagai

Supervision and writing - review and editing: Norihiro Itsubo

### Approval by Institutional Review Board (IRB)

In our judgment, there was no need to seek formal ethics clearance because the project did not collect personal data from patients or healthcare providers or expose either group to any risk; hence an ethics approval was not sought.

### Data Availability

Data are available upon request to the corresponding author. The rights for the IDEA prohibited the description of specific values for basic units. Industrial waste was applied instead of clinical waste due to the lack of a specific inventory in Japan.
